# Alexithymia and fibromyalgia: clinical evidence

**DOI:** 10.3389/fpsyg.2013.00909

**Published:** 2013-12-02

**Authors:** Marialaura Di Tella, Lorys Castelli

**Affiliations:** Department of Psychology, University of TurinTurin, Italy

**Keywords:** fibromyalgia, alexithymia, emotional dysregulation, depression, anxiety

## Abstract

This review proposes a critical discussion of the latest studies investigating the presence of alexithymia in patients with fibromyalgia (FM) and its relation to other psychological disorders. The focus is on the most relevant literature exploring the relationship between FM, a chronic pain syndrome, and alexithymia, an affective dysregulation, largely observed in psychosomatic diseases. The articles were selected from the Medline/Pubmed database using the search terms “Fibromyalgia,” “Alexithymia,” and “Psychological Distress.” Of the seven studies fulfilling these criteria, one found no differences between FM patients and the control group, four found significant differences, with higher levels of alexithymia in the FM sample, while two showed unclear results. Overall, the majority of findings highlighted the high prevalence of alexithymia in FM patients. Future studies should clarify the role of alexithymia in FM, paying attention to two principal aspects: the use, as a control group, of patients with chronic pain conditions but a low psychosomatic component, and the use of other measures, in addition to the Toronto Alexithymia Scale (TAS-20), to assess alexithymia.

## INTRODUCTION

The main aim of this review is to propose a critical discussion of the most recent studies investigating the presence of alexithymia in patients affected by fibromyalgia (FM) syndrome, and its relation to psychological disorders, frequently observed in these patients.

Fibromyalgia is a chronic syndrome characterized by widespread musculoskeletal pain, with an unknown etiology (Wolfe et al., 2010), while alexithymia is an affective dysregulation, characterized by difficulties in identifying and communicating feelings, and externally oriented thinking (Taylor, 1984).

Using the keywords “Fibromyalgia,” “Alexithymia,” and “Psychological Distress,” the Medline/Pubmed database was searched for studies examining the prevalence of alexithymia in FM patients published up to October 2012 which were then analyzed. In particular, target articles were searched using the following associations: fibromyalgia AND alexithymia, fibromyalgia AND psychological distress, fibromyalgia AND alexithymia AND psychological distress. Abstracts were checked and reviews, comments, and studies not concerning the relationship between fibromyalgia, alexithymia, and psychological distress were excluded. Seven studies were ultimately included in this review.

## FIBROMYALGIA SYNDROME

Fibromyalgia is a syndrome characterized by chronic, widespread musculoskeletal pain with tenderness over specific trigger points (Mease, 2005; Mease et al., 2009). Its prevalence is estimated to be between 3 and 6% of the world population (WHO, 2008), predominantly in women, with a female to male ratio of 10:1 (Macfarlane et al., 1999; Anderberg et al., 2000). It is most commonly diagnosed between the ages of 20 and 50, though onset can occur in childhood.

Fibromyalgia symptoms are not restricted to pain, but often include a heterogeneous series of other conditions, such as hyperalgesia and/or allodynia, physical and mental fatigue, disrupted or non-restorative sleep, headaches, irritable bowels, cognitive impairments, and other functional complaints (Mease, 2005), in the absence of laboratory or radiologic examinations to support them (Abeles et al., 2007). The etiology of this syndrome is not completely understood, but clinicians and researchers believe that it could result from different factors, such as stress, medical illness, pain conditions, and a variety of neurotransmitter and neuroendocrine disturbances (Mease, 2005).In particular, high levels of stress and psychiatric symptoms may negatively influence the perception of disease severity (Hawley et al., 1988), functional ability (Ledingham et al., 1993), and pain threshold and tolerance (Epstein et al., 1995). Some authors suggest that the development of FM might stem from stress-induced disruption of the hypothalamic-pituitary-adrenal (HPA) axis (McBeth et al., 2005). Exposure to prolonged stressful conditions can indeed alter the function of the HPA axis, with consequent increased production of the corticotrophin-releasing factor (CRF) and a potentially amplified pain perception. For this reason, FM is often defined a “*central sensitization syndrome*,” caused by increased sensitivity in the central nervous system to pain signals (Ngian et al., 2011).

Depressive and anxiety disorders (Fietta et al., 2007), as well as elevated levels of alexithymia (Sayar et al., 2004; Pedrosa-Gil et al., 2008; Huber et al., 2009; Castelli et al., 2012), have been reported, among psychological factors, as features largely present in FM syndrome.

## ALEXITHYMIA

Alexithymia is a personality dimension characterized by different cognitive and emotional features, observed in many clinical conditions, especially in psychosomatic disorders (Taylor, 2000). Sifneos (1972) coined the term alexithymia, literally “absence of words for emotion,” to describe people who lack the ability to communicate their feelings or have limited imagination (Lesser, 1985). The main aspects of alexithymia, which can be considered a deficit in cognitive processing and emotions regulation (Kooiman et al., 2004), are a difficulty in identifying and describing subjective feelings, a difficulty distinguishing between feelings and the bodily sensations of emotional arousal, restricted imagination processes, as evidenced by a lack of imagination, and a stimulus-bound, externally oriented cognitive style (Taylor et al., 1997). Alexithymic individuals are therefore incapable of adequately identifying physical sensations such as the somatic manifestations of emotions and may tend to misinterpret their emotional arousal as signs of disease (Lumley et al., 1996). This makes them susceptible to incorrectly attributing emotion-related physical symptoms to physical disease and to seeking medical care for symptoms for which no medical explanations can be found (Tuzer et al., 2011).

The presence of alexithymic traits can be assessed with different instruments such as observer-rated measures, including the Observer Alexithymia Scale (Haviland et al., 2000) and the Toronto Structured interview for Alexithymia (Bagby et al., 2006), or self-report measures, including the Levels of Emotional Awareness Scale (LEAS; Lane and Schwartz, 1987) to evaluate written emotional response, or the 20-item Toronto Alexithymia Scale (TAS-20; Bagby et al., 1994a,b).

Of these different instruments, the TAS-20 is one of the most commonly used by clinicians and researchers, and was the principal method employed to assess alexithymia in the studies analyzed. It provides investigators with a reliable, validated, and common metric for measuring the construct (Taylor and Bagby, 2004; Lumley et al., 2007). The TAS-20 consists of three subscales: *difficulty identifying feelings*(F1), which measures the inability to distinguish between specific emotions, or between emotions and the bodily sensations of emotional arousal; *difficulty describing feelings*(F2), which assesses the inability to verbalize one’s emotions to others; and *externally oriented thinking*(F3), which evaluates the tendency of individuals to focus their attention externally and not on the inner emotional experience (Taylor and Bagby, 2004; Lumley et al., 2007).

## FIBROMYALGIA SYNDROME AND ALEXITHYMIA

Psychiatric disorders and psychological distress are some of the predominant features of FM, which may contribute to the manifestation, retention, and intensification of the symptomatology (Fietta et al., 2007; Aguglia et al., 2011).

Among the psychological factors, alexithymia is a personality construct which has been minimally explored in FM but is worth examining, as it can interfere with the perception of emotional sensations.

The inability to emotionally regulate, especially negative feelings, is thought to result in increased negative affects, chronic sympathetic hyperarousal, and impaired immune status, which can lead to the development or exacerbation of somatic disease and pain (Lumley et al., 1996; Huber et al., 2009). On the other hand, alexithymia could promote maladaptive illness behaviors, defined as a patient’s ideas, affects, attitudes, and behaviors in relation to illness and the sick role, since alexithymic individuals may focus on, amplify, or overreact to unpleasant physical sensations (Pilowsky and Katsikitis, 1994).

To date, different studies have investigated the presence of alexithymia in FM patients, using distinct experimental procedures. Of the seven studies selected for the current review, three assessed alexithymia rates comparing FM patients to patients with other chronic pain conditions, one compared alexithymia levels comparing FM patients to healthy controls and three evaluated the prevalence of alexithymia comparing FM patients’ values to normative data from healthy populations. In all these studies, the assessment of alexithymia was based on the use of TAS-20 (see **Table 1**).

**Table 1:** Examined studies concerning the multivariate relationship between FM syndrome and alexithymia (TAS-20).

Study	Subjects	Results
**Patients with other chronic pain conditions as control group (three studies)**
Sayar et al. (2004)	50 FM patients	Significant higher alexithymia scores (total and factor 1 scores) in FM compared to both control groups.
	20 rheumatoid arthritis patients
	42 healthy controls
Steinweg et al. (2011)	48 FM patients	44% of alexithymia in FM group vs. 8% in general medicine group and 21% in rheumatoid arthritis group. Alexithymia was strongly associated with moderate to severe depression.
	36 general medicine patients
	43 rheumatoid arthritis patients
Tuzer et al. (2011)	70 FM patients	Significant higher alexithymia scores in FM sample compared to both CLBP and healthy control groups.
	56 chronic low back pain (CLBP) patients
	72 healthy controls
**Healthy controls (one study)**
Malt et al. (2002)	42 FM patients	No differences in alexithymia scores between FM group and healthy controls.
	48 healthy controls
**No control group (three studies)**
Pedrosa-Gil et al. (2008)	40 FM patients	15% of alexithymia in FM group.
Huber et al. (2009)	68 FM patients	Significant differences only on the 1 factor of TAS-20.
Castelli et al. (2012)	55 FM patients	20% of alexithymia in FM group.

The studies listed in the table obtained, at least in part, distinct results. In particular, one study did not find differences between FM patients and the control group (Malt et al., 2002), four found significant differences (Sayar et al., 2004; Pedrosa-Gil et al., 2008; Huber et al., 2009; Castelli et al., 2012), and two found unclear results (Steinweg et al., 2011; Tuzer et al., 2011).

Malt et al. (2002) found no differences in alexithymia scores in a group of 42 female FM patients compared with an age-matched random sample of female healthy controls and did not report data on different subscales scores of TAS-20. A high positive correlation was detected between alexithymia scores and scores for anxiety, depression, and neuroticism, allowing the authors to suppose that alexithymia could be associated with psychological distress.

On the other hand, Sayar et al. (2004), found significantly higher alexithymia scores and more difficulties in identifying feelings among FM patients with respect to rheumatoid arthritis patients or healthy controls. Moreover, the rate of alexithymia did not change when the severity of pain or the level of depression were controlled for FM patients, showing that alexithymia in the FM sample examined was independent of depression or pain. Pedrosa-Gil et al. (2008) also found a higher prevalence of alexithymia in a sample of female FM patients (15%) compared to values observed in non-clinical populations (7.9%; Posse et al., 2002). In addition, they highlighted the presence of a possible relation between dysfunctional emotional regulation, the core feature of alexithymia, and problematic early relationships in the parental bonding process in FM patients. Whereas Huber et al. (2009) found that a significant difference was present between the sample of female FM patients recruited and the Italian healthy normative data (see Bressi et al., 1996), but only on the “difficulty identifying feeling” (DIF) subscale of the TAS-20. In addition, alexithymia – the DIF subscale in particular – was associated with increased affective pain and hypochondriacal illness behavior.

In line with other evidence (Lumley et al., 1996, 2007), the authors considered the former relationship to be the result of an excessive focus of alexithymics on their body or a tendency to misinterpret bodily sensations of emotional arousal as symptoms of physical illness and thought it could be mediated by psychological distress and illness behavior. Similarly, Castelli et al. (2012) reported a higher prevalence of alexithymia in a female FM sample (20%) with respect to the general population (see above); in this case, too, the difference was mainly due to difficulties in identifying feelings in FM patients, whereas the other subcomponents of the TAS-20, “difficulty describing feeling” and “externally oriented thinking,” were not significantly different from normative data.

Of the three studies that enrolled a sample of patients affected by chronic pain conditions, without a psychosomatic component [i.e., rheumatoid arthritis and chronic low back pain (CLBP)], Steinweg et al. (2011) found that the prevalence of alexithymia in FM patients was significantly higher than in either the general medicine group or the rheumatoid arthritis group. However, the higher level of alexithymia seen in the FM sample appeared only after the presence of moderate to severe depression, since the differences in alexithymia scores became insignificant when corrected for depression rate. Likewise, Tuzer et al. (2011) found that alexithymia, as well as somatization, depression, anxiety, and hostility, was significantly higher in a female FM sample compared with a CLBP group or healthy control group. Yet considering that difficulty in identifying and describing feelings reciprocally predicted depression and anxiety symptoms in the study, it is not clear whether alexithymia is a secondary defensive reaction against negative affects or a personality trait related to emotional trauma in women (Lumley and Sielky, 2000; De Gucht and Heiser, 2003).

The results, at least partially contrasting, obtained in the studies mentioned above, might depend on different reasons. One could be related to the substantial individual differences in pain severity and disability in patients with persistent pain (Sayar et al., 2004). In most cases, pain in FM is rated as more severe than in other chronic pain conditions (Walter et al., 1998). It is therefore important to compare FM patients with other chronic pain groups, after controlling for pain severity, since the differences in alexithymia levels may be influenced by variations in the experience of pain and disability (Lumley et al., 2002).

Another possible reason could be the exclusive use of the TAS-20 for the assessment of alexithymia. In recent years, this instrument has been criticized for its inability to detect the most severe cases of alexithymia, the difficulty in replicating its factor structure, and its association with negative affectivity (Lane et al., 1996; Kooiman et al., 2002; Baiardini et al., 2011). Furthermore, the use of explicit self-reports as an exclusive measure to assess alexithymia has been questioned. Explicit self-reports require respondents to be aware of their lack of emotional awareness and reduced capacity to describe and identifying feelings (Parling et al., 2010). A better way to understand the construct could be the parallel use of a performance-based instrument (see for example the LEAS; Lane et al., 1990) or a structured interview, where individuals are not required to assess their own emotional abilities alone and the examiner determines the appropriate levels of emotional awareness through their descriptions (Baeza-Velasco et al., 2012).

## ALEXITHYMIA AND PSYCHOLOGICAL DISTRESS IN FIBROMYALGIA SYNDROME

The presence of psychological distress, especially depression and anxiety, is a well-known aspect of FM syndrome and has been reported in many studies (Fietta et al., 2007). At the same time, the increased prevalence of alexithymia in patients with depression is now well-established (Honkalampi et al., 2000). It is therefore important to clarify whether or not the prevalence of alexithymia in FM patients can be accounted for simply by the presence of depressive symptoms. In the studies discussed above, the role of psychological distress, in particular depression, is highlighted in mediating the relationship between FM and alexithymia. As mentioned before, Steinweg et al. (2011) found that the higher levels of alexithymia seen in FM patients were due only to the presence of moderate to severe depression, since the differences in alexithymia scores became insignificant when controlled for depression rate. On the other hand, Castelli et al. (2012), studying the quality of life in FM patients found, in both regression analyses performed, that when the psychological distress variables were added as competing predictors, alexithymia ceased to be significant in explaining the health-related quality of life variance. A possible interpretation, given by the authors, is that the inability of individuals with high degrees of alexithymia to identify accurately their own feelings limits not only their ability to regulate their emotions, but also verbal communication of psychological distress, with a potentially negative impact on depression and anxiety levels (Taylor et al., 1997). Similarly, investigating the types of causal attributions in women with chronic pain, Tuzer et al. (2011) found that the significant relationship between alexithymia scores, the number of somatic symptoms, and psychological attributions, in the FM group, became insignificant when the effects of psychological distress variables were controlled for. In other words, the former association appeared to be caused simply by the effects of depression and anxiety on the FM patients.

These results reveal the strong link between alexithymia and psychological distress in FM syndrome, especially depression. So it is essential for further research to consider the impact of alexithymia trait and its possible relationship with psychological distress and psychiatric disorders.

## CONCLUSION

Fibromyalgia is a multifaceted condition in which many factors are involved in the onset and retention of the disease. Psychological features are some of the principal aspects that contribute to the disability caused by this pathology and it is therefore essential to investigate them. Alexithymia is an emotional dysregulation trait, largely observed in psychosomatic disorders (Taylor, 2000) that could play an important role in FM syndrome. Alexithymic individuals are, in fact, incapable of adequately identifying physical sensations such as the somatic manifestations of emotions and may tend to misinterpret their emotional arousal as signs of disease (Lumley et al., 1996). This could amplify the perception of disease, bringing patients to seek medical care for symptoms for which there is no medical explanation.

The available studies show the high prevalence of alexithymic traits in FM patients, ranging from 15 to 20% (Pedrosa-Gil et al., 2008; Castelli et al., 2012). However, the unclear results found in some of these works indicate a need to shed light on the intensity and unambiguousness of this relationship. As mentioned before, there are two possible strategies for reaching this goal. One could be to use a performance-based instrument (see for example the LEAS; Lane et al., 1990) or a structured interview, in addition to commonly employed self-report measures (such as the TAS-20), for the assessment of alexithymia. The use of explicit self-reports requires, in fact, the respondents to be aware of their emotional dysregulation (Parling et al., 2010). The use of a performance-based instrument or a structured interview could represent a step forward toward a better understanding of this phenomena (Baeza-Velasco et al., 2012).

The other possibility is to use a control group of patients with chronic pain conditions with a low psychosomatic component (such as rheumatoid arthritis patients). The differences observed in alexithymia levels may be influenced by variations in the experience of pain, usually rated as more severe in FM syndrome than in other chronic pain diseases (Walter et al., 1998). So to better understand the prevalence of this psychological dimension in FM syndrome, it is important to compare FM patients with other chronic pain groups, after controlling for pain severity. An additional issue for further studies would be to analyze potential differences in the evolution of FM syndrome, with reference to the presence or absence of alexithymia. Specifically, it would be useful to investigate whether and how alexithymia influences the psychopathological symptoms, and the compliance to psychological and psychopharmacological interventions. Clarifying the role of alexithymia in FM can thus allow better understanding of the disease etiology and the development of patient-tailored treatments, in which both physical and psychological symptoms have to be considered.

## Conflict of Interest Statement

The authors declare that the research was conducted in the absence of any commercial or financial relationships that could be construed as a potential conflict of interest.
